# Hemodynamic parameters to guide fluid therapy

**DOI:** 10.1186/2110-5820-1-1

**Published:** 2011-03-21

**Authors:** Paul E Marik, Xavier Monnet, Jean-Louis Teboul

**Affiliations:** 1 Department of Medicine, Division of Pulmonary and Critical Care Medicine, Eastern Virginia Medical School, Norfolk, VA, USA; 2 Medical Intensive Care Unit, Research Unit EA 4046, Bicetre Teaching Hospital, Paris-11 University, Le Kremlin-Bicetre, France

## Abstract

The clinical determination of the intravascular volume can be extremely difficult in critically ill and injured patients as well as those undergoing major surgery. This is problematic because fluid loading is considered the first step in the resuscitation of hemodynamically unstable patients. Yet, multiple studies have demonstrated that only approximately 50% of hemodynamically unstable patients in the intensive care unit and operating room respond to a fluid challenge. Whereas under-resuscitation results in inadequate organ perfusion, accumulating data suggest that over-resuscitation increases the morbidity and mortality of critically ill patients. Cardiac filling pressures, including the central venous pressure and pulmonary artery occlusion pressure, have been traditionally used to guide fluid management. However, studies performed during the past 30 years have demonstrated that cardiac filling pressures are unable to predict fluid responsiveness. During the past decade, a number of dynamic tests of volume responsiveness have been reported. These tests dynamically monitor the change in stroke volume after a maneuver that increases or decreases venous return (preload) and challenges the patients' Frank-Starling curve. These dynamic tests use the change in stroke volume during mechanical ventilation or after a passive leg raising maneuver to assess fluid responsiveness. The stroke volume is measured continuously and in real-time by minimally invasive or noninvasive technologies, including Doppler methods, pulse contour analysis, and bioreactance.

## Introduction

The cornerstone of treating patients with shock remains as it has for decades: intravenous fluids. Surprisingly, dosing intravenous fluid during resuscitation of shock remains largely empirical. Too little fluid may result in tissue hypoperfusion and worsen organ dysfunction; however, over-prescription of fluid also appears to impede oxygen delivery and compromise patient outcome. Recent data suggest that early aggressive resuscitation of critically ill patients may limit and/or reverse tissue hypoxia, progression to organ failure, and improve outcome [1]. In a landmark study, Rivers et al. demonstrated that a protocol of early goal-directed therapy reduces organ failure and improves survival in patients with severe sepsis and septic shock [2]. Similarly, a protocol to optimize preload and cardiac output in patients undergoing major surgery reduced postoperative complications and length of stay [3]. Uncorrected hypovolemia, leading to inappropriate infusions of vasopressor agents may increase organ hypoperfusion and ischemia [4]. However, overzealous fluid resuscitation has been associated with increased complications, increased length of intensive care unit (ICU) and hospital stay, and increased mortality. A review of the ARDSNet cohort demonstrated a clear positive association between the mean cumulative daily fluid balance and mortality [5]. Murphy and colleagues demonstrated a similar finding in patients with septic shock [6]. Data from the "VAsopressin in Septic Shock Trial" demonstrated that the quartile of patients with the highest fluid balance at both 12 hours and 4 days had the highest adjusted mortality [7]. The extravascular lung water index (EVLWI) can be accurately "measured" in critically ill patients by using the single indicator transpulmonary thermodilution method) [8,9]. The EVLWI appears to be a useful tool to quantify "capillary leakiness" and the degree of interstitial edema [10]. A number of authors have demonstrated a positive relationship between EVLWI and mortality in critically ill patients [11,12].

The first step in the hemodynamic management of critically ill patients is to determine the adequacy of tissue/organ perfusion. Although the signs of shock may be obvious, those of subclinical hypoperfusion may be more subtle (Table 1). It should be noted that increasing cardiac output/oxygen delivery in patients with adequate organ perfusion serves no useful purpose. Indeed, studies of yesteryear have demonstrated that targeting "supra-normal" hemodynamic parameters may be harmful [13,14]. In those patients with indices of inadequate tissue perfusion, fluid resuscitation is generally regarded as the first step in resuscitation. Clinical studies have, however, demonstrated that only approximately 50% of hemodynamically unstable critically ill patients are volume-responsive [15]. The resuscitation of the critically ill patient requires an accurate assessment of the patient's intravascular volume status (cardiac preload) and the likelihood that the patient will respond (increase stroke volume) to a fluid challenge (volume responsiveness). Patients with overt shock and those with subclinical shock and who are fluid-responsive are best managed by 500 to 1,000 mL boluses of fluid. Fluid boluses should be discontinued once the patient is no longer fluid-responsive or the EVLWI has increased significantly.

**Table 1:** Clinical indices of the adequacy of tissue/organ perfusion

• Mean arterial pressure
Cerebral and abdominal perfusion pressures
• Urine output
• Mentation
• Capillary refill
• Skin perfusion/mottling
• Cold extremities (and cold knees)
• Blood lactate
• Arterial pH, BE, and HCO3
• Mixed venous oxygen saturation SmvO_2 _(or ScvO_2_)
• Mixed venous pCO_2_
• Tissue pCO_2_
• Skeletal muscle tissue oxygenation (StO_2_)

**Figure 1:**
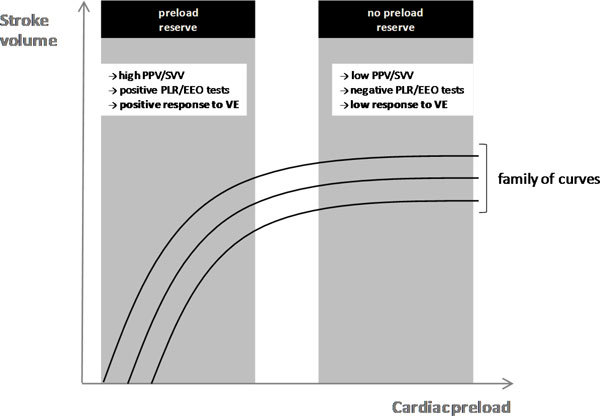
**Frank-Starling relationship**. Once the ventricle is functioning on the steep part of the Frank-Starling curve, there is a preload reserve. Volume expansion (VE) induces a significant increase in stroke volume. The pulse pressure (PPV) and stroke volume (SVV) variations are marked and the passive leg raising (PLR) and end-expiratory occlusion (EEO) tests are positive. By contrast, once the ventricle is operating near the flat part of the curve, there is no preload reserve and fluid infusion has little effect on the stroke volume. There is a family of Frank-Starling curves depending upon the ventricular contractility.

Fundamentally, the only reason to give a patient a fluid challenge is to increase stroke volume (volume responsiveness). If the fluid challenge does not increase stroke volume, volume loading serves the patient no useful benefit (may be harmful). According to the Frank-Starling principle, as the preload increases left ventricular (LV) stroke volume increases until the optimal preload is achieved at which point the stroke volume remains relatively constant (Figure 1). This optimal preload is related to the maximal overlap of the actin-myosin myofibrils. It is important to note that in an intact heart the actin-myosin links cannot be disengaged and hence there is no descending limb of the Frank-Starling curve. Once the left ventricle is functioning near the "flat" part of the Frank-Starling curve, fluid loading has little effect on the stroke volume. In normal physiologic conditions, both ventricles operate on the ascending portion of the Frank-Starling curve [16]. This mechanism provides a functional reserve to the heart in situations of acute stress. In normal individuals, an increase in preload (with volume challenge) results in a significant increase in stroke volume [17] (Figure 1).

Traditionally the central venous pressure (CVP) has been used to guide fluid management. A European survey of intensivists/anesthesiologists reported that more than 90% used the CVP to guide fluid management [18]. A Canadian survey reported that 90% of intensivists use the CVP to monitor fluid resuscitation in patients with septic shock [19]. The basis for using the CVP to guide fluid management comes from the misguided dogma that the CVP reflects intravascular volume; specifically, it is widely believed that patients with a low CVP are volume-depleted, whereas patients with a high CVP are volume-overloaded. Furthermore, the "5-2" rule, which was popularized in the 1970 s [20], is still widely used today for guiding fluid therapy. According to this rule, the change in CVP after a fluid challenge is used to guide subsequent fluid management decisions. The CVP is a good approximation of right atrial pressure, which is a major determinant of right ventricular (RV) filling. It has been assumed that the CVP is a good indicator of RV preload. Furthermore, because RV stroke volume determines LV filling, the CVP is assumed to be an indirect measure of LV preload. However, due to the changes in venous tone, intrathoracic pressures, LV and RV compliance, and geometry that occur in critically ill patients, there is a poor relationship between the CVP and RV end-diastolic volume. Furthermore, the RV end-diastolic volume may not reflect the patients' position on the Frank-Starling curve and therefore his/her preload reserve. More than 100 studies have been published to date that have demonstrated no relationship between the CVP (or change in CVP) and fluid responsiveness in various clinical settings [21]. Indeed, there have only been two studies published in the world literature that demonstrate "some relationship" between the CVP and intravascular volume; both of these studies were conducted in standing horses [22,23]! Based on this information, we believe that the CVP should no longer be routinely used for guiding fluid management in the ICU, operating room, or emergency room.

Until recently, it has been unclear as to which hemodynamically unstable patients are volume-responsive and likely to benefit from fluid resuscitation (fluid boluses). However, during the past decade a number of dynamic tests of volume responsiveness have been reported. These tests dynamically monitor the change in stroke volume after a maneuver that increases or decreases venous return (preload). These tests allow the clinician to determine the individual patient's position on his/her Frank-Starling curve, and thus determine whether the patient is likely to be fluid-responsive. These techniques use the change in stroke volume during mechanical ventilation or after a passive leg raising (PLR) maneuver to assess fluid responsiveness.

## Heart-lung interactions during mechanical ventilation

### Dynamic changes in stroke volume, pulse pressure, and oximetric waveform

**Figure 2:**
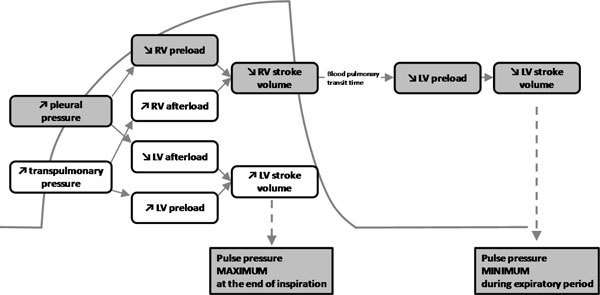
**Heart-lung interactions**. Hemodynamic effects of mechanical ventilation. The cyclic changes in left ventricular (LV) stroke volume are mainly related to the expiratory decrease in LV preload due to the inspiratory decrease in right ventricular (RV) filling. Reproduced with permission from Critical Care/Current Science Ltd [24].

An impressive number of studies have demonstrated that the pulse pressure variation (PPV) derived from analysis of the arterial waveform, the stroke volume variation (SVV) derived from pulse contour analysis, and the variation of the amplitude of the pulse oximeter plethysmographic waveform to be highly predictive of fluid responsiveness [15]. The principles underling this technique are based on simple physiology (Figure 2). Intermittent positive-pressure ventilation induces cyclic changes in the loading conditions of the left and right ventricles. Mechanical insufflation decreases preload and increases afterload of the right ventricle. The RV preload reduction is due to the decrease in the venous return pressure gradient that is related in the inspiratory increase in pleural pressure [24]. The increase in RV afterload is related to the inspiratory increase in transpulmonary pressure. The reduction in RV preload and increase in RV afterload both lead to a decrease in RV stroke volume, which is at a minimum at the end of the inspiratory period [25]. The inspiratory reduction in RV ejection leads to a decrease in LV filling after a phase lag of two or three heart beats because of the long blood pulmonary transit time. Thus, the LV preload reduction may induce a decrease in LV stroke volume, which is at its minimum during the expiratory period when conventional mechanical ventilation is used. The cyclic changes in RV and LV stroke volume are greater when the ventricles operate on the steep rather than the flat portion of the Frank-Starling curve (Figure 1). The magnitude of the respiratory changes in LV stroke volume is an indicator of biventricular preload dependence [24]. With remarkable consistency, a variation of greater than 12% to 13% has been reported to be highly predictive of volume responsiveness (Table 2) [15].

**Table 2:** Predictive value of techniques used to determine fluid responsiveness [15]

Method	Technology	AUC*
Pulse pressure variation (PPV)	Arterial waveform	0.94 (0.93-0.95)
Systolic pressure variation (SPV)	Arterial waveform	0.86 (0.82-0.90)
Stroke volume variation (SVV)	Pulse contour analysis	0.84 (0.78-0.88)
Left ventricular end-diastolic area (LVEDA)	Echocardiography	0.64 (0.53-0.74)
Global end-diastolic volume (GEDV)	Transpulmonary thermodilution	0.56 (0.37-0.67)
Central venous pressure (CVP)	Central venous catheter	0.55 (0.48-0.62)

*AUC = area under the curve with 95% confidence intervals

The pulse oximeter plethysmographic waveform differs from the arterial pressure waveform by measuring volume rather than pressure changes in both arterial and venous vessels. As an extension of pulse pressure analysis during mechanical ventilation, dynamic changes in both the peak and amplitude of the pulse oximeter plethysmographic waveform have been used to predict fluid responsiveness [26]. The dynamic changes of the plethysmographic waveform with positive pressure ventilation have shown a significant correlation and good agreement with the PPV and have accurately predicted fluid responsiveness in both the operating room and ICU setting [27-29]. The "Pleth Variability Index" (PVI) is an automated measure of the dynamic change in the "Perfusion Index" that occurs during a respiratory cycle (Masimo Corporation, Irvine, CA). The "Perfusion Index" is the infrared pulsatile signal indexed against the nonpulsatile signal and reflects the amplitude of the pulse oximeter waveform. The PVI correlates closely with the respiratory induced variation in the plethysmographic and arterial pressure waveforms and can predict fluid responsiveness noninvasively in mechanically ventilated patients [30,31]. These oximetric techniques may be valuable for monitoring fluid responsiveness in ICU and surgical patients who do not have an arterial catheter *in situ*.

It should be appreciated that both arrhythmias and spontaneous breathing activity will lead to misinterpretations of the respiratory variations in pulse pressure/stroke volume. Furthermore, for any specific preload condition the PPV/SVV will vary according to the tidal volume. Reuter and colleagues demonstrated a linear relationship between tidal volume and SVV [32]. De Backer and colleagues evaluated the influence of tidal volume on the ability of the PPV to predict fluid responsiveness [33]. These authors reported that the PPV was a reliable predictor of fluid responsiveness only when the tidal volume was at least 8 mL/kg. For accuracy, reproducibility, and consistency we suggest that the tidal volume be increased to 8 to 10 mL/kg ideal body weight before and after a fluid challenge.

### Dynamic changes in aortic flow velocity/stroke volume assessed by Doppler methods

The respiratory changes in aortic flow velocity and stroke volume can be assessed by Doppler echocardiography. Assuming that the aortic annulus diameter is constant over the respiratory cycle, the changes in aortic blood velocity should reflect changes in LV stroke volume. Feissel and colleagues demonstrated that the respiratory changes in aortic blood velocity as measured by transesophageal echocardiography predicted fluid responsiveness in mechanically ventilated patients [34]. Similarly, ventilator-induced variation in descending aortic blood flow measured by esophageal Doppler monitoring has been demonstrated to predict fluid responsiveness [35].

### Positive pressure ventilation induced changes in vena-caval diameter

Cyclic changes in superior and inferior vena-caval diameter as measured by echocardiography have been used to predict fluid responsiveness. Barbier and colleagues and Feissel and coworkers demonstrated that the distensibility index of the inferior vena cava, which reflects the increase in the inferior vena cava diameter on inspiration (mechanical ventilation), was able to predict fluid responsiveness [36,37]. Similarly, Vieillard-Baron and colleagues have demonstrated that the collapsibility index of the superior vena cava is highly predictive of volume responsiveness [38,39]. Both of these techniques are not conducive to continuous monitoring. Furthermore, the superior vena cava can only be adequately visualized by transesophageal echocardiography.

### The end-expiratory occlusion test

**Figure 3:**
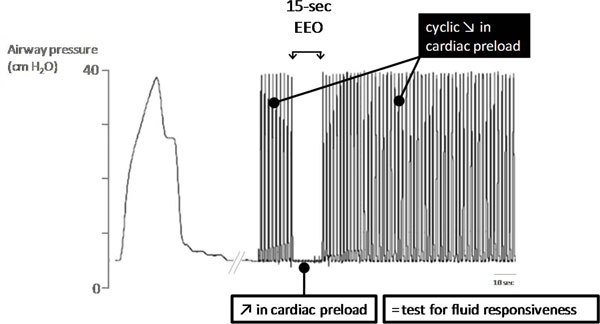
**End-expiratory occlusion test**. The end-expiratory occlusion (EEO) test consists in interrupting mechanical ventilation at the end of expiration during 15 seconds. This suppresses the cyclic decrease in cardiac preload, which normally occurs at each mechanical insufflation. Therefore, this brief procedure should increase cardiac preload and can serve as a test to assess preload responsiveness and hence to predict the response to a subsequent fluid infusion.

During mechanical ventilation, each insufflation increases intrathoracic pressure and thus reduces the systemic venous return. In a recent study, it was hypothesized that interrupting mechanical insufflation during an end-expiratory occlusion can increase cardiac preload sufficiently for such a test being used to predict fluid responsiveness [40] (Figure 3). This study included 34 patients who received mechanical ventilation. Patients had cardiac arrhythmias or exhibited a certain degree of spontaneous breathing activity not sufficient to interrupt the brief (15 seconds) end-expiratory occlusion of the respiratory circuit. In patients who will increase their cardiac output by more than 15% in response to a subsequent 500-mL fluid challenge (fluid responders), the end-expiratory occlusion test increased both arterial pulse pressure and pulse contour cardiac output [40]. In nonresponder patients, the end-expiratory occlusion test did not induce a significant increase in pulse pressure and cardiac output. An increase in cardiac output and arterial pulse pressure by more than 5% predicted fluid responsiveness with a good accuracy. This test is easy to use in clinical practice and seems to be reliable in cases of cardiac arrhythmias and of low tidal volume, conditions where PPV and SVV are unreliable [X Monnet and JL Teboul, personal observations].

## Passive leg raising

**Figure 4:**
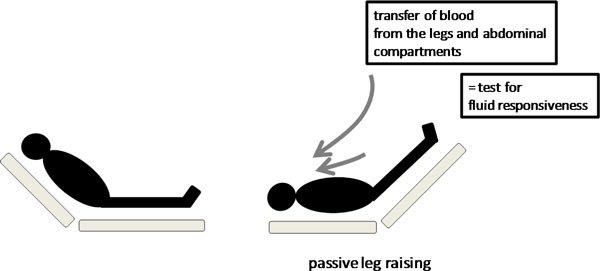
**Passive leg raising**. The passive leg raising test consists in measuring the hemodynamic effects of a leg elevation up to 45°. A simple way to perform the postural maneuver is to transfer the patient from the semirecumbent posture to the passive leg raising position by using the automatic motion of the bed.

In the initial stages of resuscitation in the emergency room, ward, or ICU, most patients are not intubated and are breathing spontaneously. In addition, with the reduced use of sedative agents in the ICU, many critically ill patients are ventilated with modes of ventilation that allow spontaneous breathing activity. Because the respiratory variability of hemodynamic signals cannot be used for predicting volume responsiveness in spontaneously breathing patients, other techniques, such as passive leg raising (PLR), have been proposed for this purpose [41,42]. Lifting the legs passively from the horizontal position induces a gravitational transfer of blood from the lower limbs toward the intrathoracic compartment (Figure 4). Accordingly, increases in the pulmonary artery occlusion pressure and the LV ejection time [43] have been reported during PLR, supporting the evidence that the volume of blood transferred to the heart during PLR is sufficient to increase the left cardiac preload and thus challenge the Frank-Starling curve. Beyond its ease of use, this method has the advantage of reversing its effects once the legs are tilted down [43,44]. Therefore, PLR may be considered a reversible "autotransfusion."

The concept of detecting preload responsiveness by using PLR emerged from a study in mechanically ventilated patients, where the increase in thermodilution stroke volume after a fluid infusion correlated with the increase in arterial pulse pressure produced by PLR [44]. The ability of PLR to serve as a test of preload responsiveness has been confirmed in additional studies performed in critically ill patients [40,43,45-51]. The change in aortic blood flow (measured by esophageal Doppler) during a 45° leg elevation was shown to predict the changes in aortic blood flow produced by a 500-mL fluid challenge even in patients with cardiac arrhythmias and/or spontaneous ventilator triggering, situations in which PPV lost its predictive ability [43]. A recent meta-analysis, which pooled the results of eight recent studies, confirmed the excellent value of PLR to predict fluid responsiveness in critically ill patients with a global area under the receiver operating characteristic curve of 0.95 [51]. The best way to perform a PLR maneuver to predict volume responsiveness is to elevate the lower limbs to 45° (automatic bed elevation or wedge pillow) while at the same time placing the patient in the supine from a 45° semirecumbent position (Figure 4). Starting the PLR maneuver from a total horizontal position may induce an insufficient venous blood shift to elevate significantly cardiac preload [52]. By contrast, starting PLR from a semirecumbent position induces a larger increase in cardiac preload because it induces the shift of venous blood not only from both the legs but also from the abdominal compartment [53]. In should be noted that intra-abdominal hypertension (intra-abdominal pressure > 16 mmHg) impairs venous return and reduces the ability of PLR to detect fluid responsiveness [54].

Because the maximal hemodynamic effects of PLR occur within the first minute of leg elevation [43], it is important to assess these effects with a method that is able to track changes in cardiac output or stroke volume on a real-time basis. In this regard, the response of descending aortic blood flow (measured by esophageal Doppler) to PLR [43,45], of the velocity-time integral (measured by transthoracic echocardiography) [46,47], and the femoral artery flow (measured by arterial Doppler) [50] to PLR have been demonstrated to be helpful in predicting the response to volume administration in patients with spontaneous breathing activity. Echocardiographic techniques are, however, operator-dependent and not conducive to continuous real-time monitoring. Until recently, continuous real-time cardiac output monitoring required a thermodilution pulmonary artery catheter. During the past decade, several less invasive methods have been developed. These techniques include transpulmonary thermodilution, pulse-contour analysis, and bioreactance. The PiCCO™ system (Pulsion Medical Systems, Munich, Germany) uses transpulmonary thermodilution to calibrate the pulse-contour-derived stroke volume, whereas the stroke volume derived from the FloTrac-Vigileo™ (Edward Lifesciences, Irvine, CA) device is uncalibrated. Both of these devices may be useful to determine the hemodynamic response to PLR. In this regard, an increase in "pulse contour" cardiac output by more than 10% in response to PLR has been shown to accurately predict volume responsiveness in mechanically ventilated patients with spontaneous breathing activity [40,48]. Although less invasive than pulmonary artery catheterization, these techniques are not ideally suited to resuscitation in the emergency room or ward or on initial presentation in the ICU. In these situations, the change in stoke volume after a PLR maneuver can be assessed noninvasively by bioreactance. Bioreactance cardiac output measurement is based on an analysis of relative phase shifts of an oscillating current that occurs when this current traverses the thoracic cavity. It differs from traditional bioimpedance-based systems, which rely on measured changes in signal amplitude [55]. The NICOM™ (Cheetah Medical, Portland, OR, USA) is comprised of a high-frequency (75 kHz) sine wave generator and four dual electrode "stickers" that are used to establish electrical contact with the body. The cardiac output as measured by bioreactance has been shown to be highly correlated with that measured by thermodilution and pulse contour analysis [55-57]. In a cohort of patients after elective cardiac surgery, Benomar and coauthors demonstrated that the NICOM™ system could accurately predict fluid responsiveness from changes in cardiac output during PLR [58]. The NICOM™ system has an algorithm with user prompts and an interface that rapidly facilitates the performance of a PLR maneuver.

Although the dynamic changes of the plethysmographic waveform have been demonstrated to be predictive of volume responsiveness in ventilated patients, this technology is poorly predictive of volume responsiveness in spontaneously breathing persons after a PLR challenge [59]. The hemodynamic effects of PLR must be assessed by a direct measure of cardiac output or stroke volume; assessing the PLR effects solely on the arterial pulse pressure leads to a significant number of false-negative cases [51]. This suggests that in spontaneously breathing patients, pulse pressure is not of sufficient sensitivity for detecting changes in stroke volume.

## Conclusions

A number of minimally invasive and noninvasive diagnostic tools are currently available that allow clinicians to assess volume responsiveness using dynamic procedures that challenge the patients' Frank-Starling curve. These technologies complement one another; each has a useful place in the continuum of the resuscitation process.

## Competing interests

Paul E. Marik has no financial interest in any of the products mentioned in this paper. Xavier Monnet and Jean-Louis Teboul are members of the medical advisory board of Pulsion Medical Systems.

## Authors' contributions

PEM, XM, and JLT contributed to draft the manuscript and approved the final version.
